# Dose-dependent interaction of dietary vitamin B2 and E in relation to cognitive performance: a cross-sectional study of older adults

**DOI:** 10.3389/fnut.2025.1597724

**Published:** 2025-09-10

**Authors:** Yueju Wu, Hai Zhou, Yong Cai, Wenjuan Du, Chaonian Li, Minli Sun

**Affiliations:** ^1^Department of Neurology, Binhai County People’s Hospital, Binhai Clinical College, Yangzhou University Medical College, Yancheng, China; ^2^Department of Neurosurgery, Binhai County People's Hospital, Clinical Medical College of Yangzhou University, Yancheng, China; ^3^Department of Traditional Chinese Medicine, Binhai County People's Hospital, Clinical Medical College of Yangzhou University, Yancheng, China; ^4^Department of Geriatrics, Binhai County People's Hospital, Clinical Medical College of Yangzhou University, Yancheng, China

**Keywords:** vitamin B2, vitamin E, cognitive function, nutrient interaction, NHANES

## Abstract

**Background:**

While individual associations of dietary vitamins with cognitive function have been widely studied, the combined role of vitamin B2 and E remains poorly understood. This study investigates their interactive effects on cognitive performance in older adults.

**Methods:**

In this cross-sectional analysis of 2,497 participants (age ≥60 years) from NHANES 2011–2014, cognitive function was assessed using the Digit Symbol Substitution Test (DSST), with low performance defined as DSST scores <33 (lowest quartile). Dietary intake of vitamins B2 and E was derived from two 24-h recalls. Multivariable logistic regression models adjusted for sociodemographic, lifestyle, and clinical covariates examined independent and joint associations, with interaction effects quantified using product terms.

**Results:**

Following comprehensive adjustment, elevated dietary intake of vitamin B2 [odds ratio (OR) = 0.74, 95% confidence interval (CI): 0.58–0.95] and vitamin E (OR = 0.73, 95% CI: 0.56–0.94) was independently associated with diminished odds of low cognitive function. A significant multiplicative interaction was observed (OR = 1.15, 95% CI: 1.05–1.26; *p* < 0.05). Marginal effect analyses revealed synergistic benefits between vitamins B2 and E at vitamin E intakes <18 mg/day, whereas antagonistic interaction emerged beyond this threshold. Furthermore, stratified analyses identified the strongest protective effects in the high-B2 + low-E group (OR = 0.56, 95% CI: 0.32–0.98) and the dual-high intake group (OR = 0.44, 95% CI: 0.27–0.73) compared to the dual-low intake group.

**Conclusion:**

Our findings highlight a dose-dependent interplay between vitamins B2 and E in modulating cognitive performance, advocating for dietary guidelines to prioritize nutrient interaction patterns in aging populations.

## Introduction

Cognitive decline poses a critical public health challenge as global populations age (1). Among dietary factors, antioxidant vitamins have gained interest for their dual capacity to neutralize free radicals and suppress neuroinflammation—key pathways implicated in cognitive aging (2). Vitamin B2 (riboflavin), a precursor to flavin adenine dinucleotide (FAD), sustains mitochondrial energy metabolism and glutathione recycling, mechanisms essential for neuronal redox balance (3). Vitamin E (*α*-tocopherol), a lipid-soluble antioxidant, protects neuronal membranes by halting lipid peroxidation cascades, though its efficacy may hinge on synergistic micronutrient interactions (4). While observational studies link higher intakes of vitamin B2 or E to reduced low cognitive function risk (5–8), these findings predominantly derive from single-nutrient analyses. This narrow focus overlooks nutrient interplay—an omission highlighted by recent trials where isolated high-dose vitamin regimens showed null or adverse cognitive effects in older adults with metabolic comorbidities (9). Complementing this, broader dietary patterns with high antioxidant capacity (such as the Mediterranean diet) have consistent benefits for functional health during aging (10). Similarly, dietary antioxidant capacity may have a protective effect against age-related diseases (11). This study extends this research paradigm by exploring the synergistic effects of micronutrients in such diets.

Emerging evidence underscores antioxidant synergy, yet critical gaps persist in understanding B-vitamin/antioxidant cross-talk. For example, combined *ω*-3 fatty acids, carotenoids, and vitamin E enhance working memory in cognitively intact older adults through dose-dependent mechanisms (12, 13). Vitamin B2’s role in sustaining reduced glutathione pools—a prerequisite for efficient *α*-tocopherol regeneration—further suggests potential neural protection synergies with vitamin E (14). Paradoxically, population studies specifically examining B2-E interactions remain absent, despite age-related declines in riboflavin absorption and FAD synthesis that may heighten reliance on dietary B2 to maintain antioxidant efficacy (15). Leveraging NHANES data, this study addresses this gap by investigating dose-dependent interactions between dietary vitamins B2 and E and their associations with cognitive function in older adults, providing critical evidence for nutrient-balanced dietary strategies.

## Methods

### Study population

**Figure 1:**
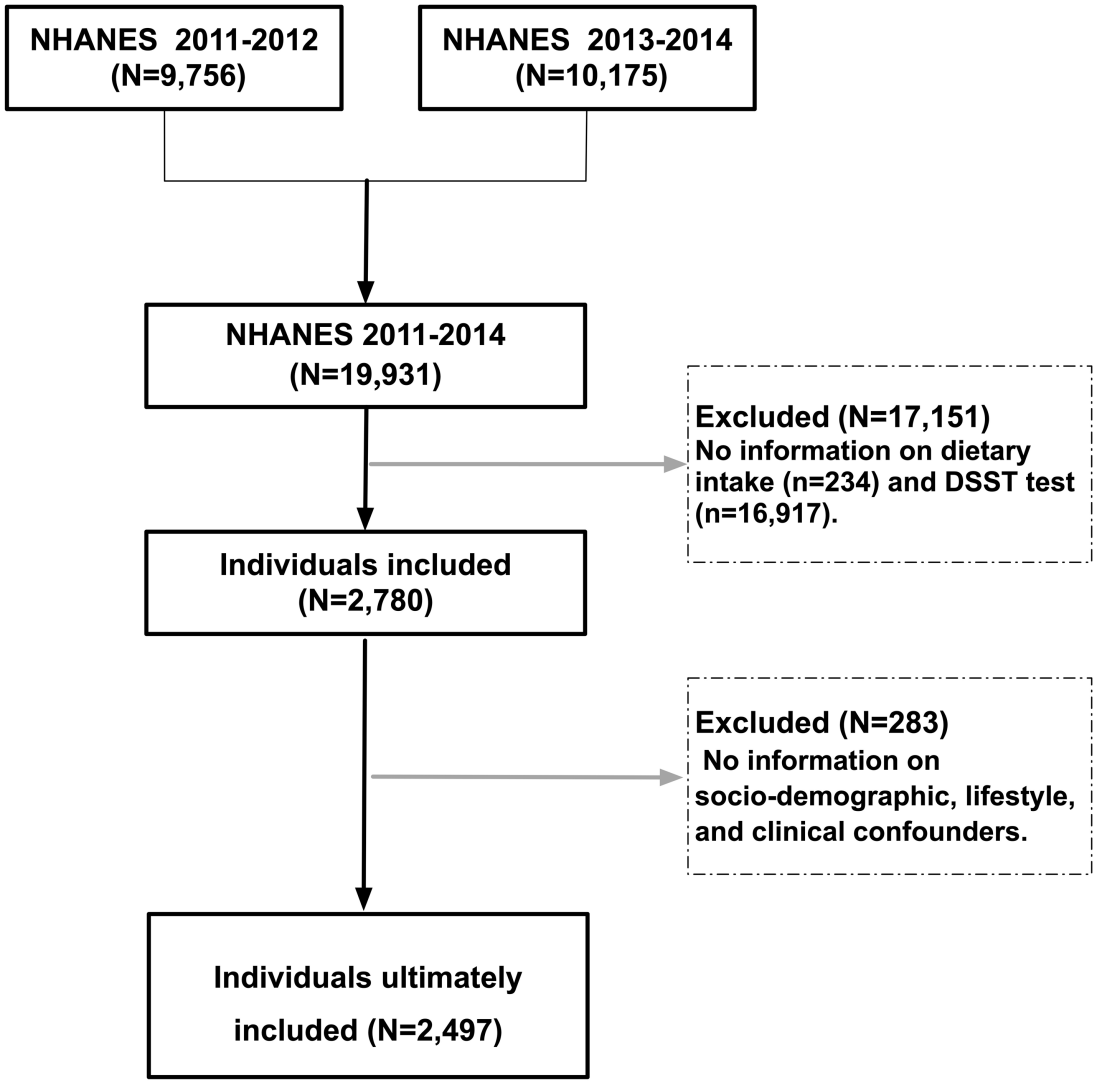
Participant selection flowchart from NHANES 2011–2014. Participants were recruited from combined NHANES 2011–2014 cycles. Exclusion criteria included incomplete dietary data (24-h recalls for vitamins B2 and E), missing cognitive function scores (Digit Symbol Substitution Test, DSST), and incomplete sociodemographic, lifestyle, or clinical covariates. A total of 2,497 older adults (age ≥60 years) met inclusion criteria for final analysis.

From NHANES 2011–2014, we included 2,497 participants aged ≥60 years after excluding those with missing dietary or covariate data (complete-case analysis). Inclusion required: (1) completion of two non-consecutive 24-h dietary recalls; (2) valid Digit Symbol Substitution Test (DSST) scores; and (3) complete sociodemographic, lifestyle, and clinical data. Analyses adhered to STROBE guidelines (16) and incorporated NHANES sampling weights to address complex survey design. Participant selection is detailed in Figure 1.

### Dietary intake and definition of low cognitive function

The dietary intake data are used to estimate the types and amounts of foods and beverages (including all types of water) consumed during the 24-h period prior to the interview (midnight to midnight) and to estimate intakes of energy, nutrients, and other food components from those foods and beverages (see the MEC In-Person Dietary Interviewers Procedures Manual for more information on the proxy interview). All employees are considered members of the organization and have undergone rigorous training. Further details can be found at: (https://wwwn.cdc.gov/Nchs/Data/Nhanes/Public/2013/DataFiles/DR2IFF_H.htm). Daily vitamin B2 and E intakes (mg/day) were averaged across two 24-h recalls. Low cognitive function was defined as DSST scores <33 (lowest quartile), consistent with prior NHANES studies (17, 18).

### Covariates

Covariates were selected based on prior evidence (19–25). Socio-demographics: Age, gender, education (<high school/high school/≥college), family poverty-to-income ratio (PIR). Lifestyle: Smoking (yes/no), alcohol use (yes/no), physical activity (sedentary/moderate/vigorous), sleep duration. Health status: BMI, diabetes (medication use or diagnosis), hypertension (medication use or diagnosis), cardiovascular disease (CVD) (including congestive heart failure, coronary artery disease, angina, myocardial infarction, or stroke) (26). Dietary factors: Total energy and fiber intake.

### Statistical analysis

Vitamin intakes were energy-adjusted using nutrient density models. Analyses proceeded in three phases: (1) Independent models assessed associations of vitamin B2 and E with cognitive function separately; (2) joint models incorporating both vitamins simultaneously; (3) Interaction models tested multiplicative effects via vitamin B2 × E product terms (continuous, per 1-SD increase). All models adjusted for sociodemographic, lifestyle, and clinical covariates. The assumptions of multivariate logistic regression analysis were fulfilled in the construction of the model. The aforementioned assumptions encompass the following: normality of the data (the distributions of continuous predictors were acceptable for maximum likelihood estimation), independence of observations (addressed using NHANES complex survey design methods), the absence of multicollinearity (all VIF values were well below the threshold of 5–10), the absence of outliers (no observations exceeded Cook’s distance >1), a linear relationship between the logarithmic odds ratio of the explanatory variables and the response variable (no significant nonlinearity was detected for continuous covariates), and sufficient sample size (the low cognitive function group included 541 cases, far exceeding the requirement of ≥10 events per variable). Interaction effects were visualized through marginal effect plots with vitamin B2 categorized as low (10th percentile), moderate (50th), or high (90th) intake. Adjusted predictions estimated marginal effects at covariate means. The 18 mg/day vitamin E interaction threshold was empirically identified using restricted cubic splines in marginal effects modeling, reflecting the inflection point where the protective association shifted from synergy to antagonism. Participants were further stratified into four groups by combined vitamin B2/E quartiles (low-B2 + low-E, low-B2 + high-E, high-B2 + low-E, high-B2 + high-E) for dose–response validation. Analyses used R v4.4.2 (survey package) with two-tailed *p* < 0.05 indicating significance.

## Results

### Population characteristics

A total of 2,497 eligible older people were included in this study, and the prevalence of low cognitive function was 21.67% (541/2,497), with a mean age of 68 (63, 74). Compared with those with normal cognitive function, those with low cognitive function tended to be older (75 *year* vs. 67 year), poorer (PIR: 1.54 vs. 3.40), less educated (<HS: 47% vs. 11%), more sedentary (60% vs. 33%), had higher rates of diabetes (31% vs. 18%), hypertension (73% vs. 57%), and CVD (37% vs. 19%), and lower intakes of energy (1,496 vs. 1,846), fiber (14 vs. 16 mg/day), vitamin B2 (1.60 vs. 1.98 mg/day), and vitamin E (5.6 vs. 7.8 mg/day) (Table 1).

**Table 1:** Baseline characteristics of study participants stratified by DSST performance.

Characteristics	Overall (*N* = 2,497)	DSST score		*p-*value
		Low (≤33)	Normal (>33)	
Age, years	68 (63, 74)	75 (67, 80)	67 (63, 73)	<0.001
BMI, (kg/m^2^)	28 (25, 32)	28 (25, 32)	28 (25, 33)	>0.9
PIR	3.13 (1.71, 5.00)	1.54 (0.98, 2.38)	3.40 (1.92, 5.00)	<0.001
Sleep duration, h	7 (6, 8)	7 (6, 8)	7 (6, 8)	0.2
Female, *n* (%)	1,272 (54%)	236 (52%)	1,036 (54%)	0.6
Education				<0.001
<HS	595 (15%)	307 (47%)	288 (11%)	
HS diploma	576 (21%)	119 (26%)	457 (21%)	
College or above	1,326 (64%)	115 (27%)	1,211 (69%)	
Smoker (current or former)	1,282 (51%)	281 (49%)	1,001 (51%)	0.6
Drinker, yes	1,885 (80%)	360 (62%)	1,525 (82%)	<0.001
Physical activity				<0.001
Sedentary	1,017 (36%)	296 (60%)	721 (33%)	
Moderate	1,004 (40%)	195 (32%)	809 (41%)	
Vigorous	476 (24%)	50 (7.7%)	426 (26%)	
Diabetes, yes	579 (19%)	176 (31%)	403 (18%)	<0.001
Hypertension, yes	1,566 (59%)	387 (73%)	1,179 (57%)	<0.001
CVD, yes	549 (21%)	169 (37%)	380 (19%)	<0.001
DSST score	53 (42, 64)	26 (21, 29)	56 (46, 66)	<0.001
Vitamin B2, mg	1.94 (1.44, 2.53)	1.60 (1.15, 2.09)	1.98 (1.48, 2.58)	<0.001
Vitamin E, mg	7.5 (5.2, 10.6)	5.6 (3.6, 8.1)	7.8 (5.4, 10.9)	<0.001
Fiber, g	16 (12, 22)	14 (9, 18)	16 (12, 22)	<0.001
Total energy, kcal	1,799 (1,422, 2,263)	1,496 (1,130, 1,898)	1,846 (1,471, 2,299)	<0.001

Low cognitive function was defined as DSST scores in the lowest population quartile (≤33). Continuous variables are presented as medians with interquartile ranges (IQR), categorical variables as frequencies. Group comparisons used Kruskal-Wallis tests for continuous variables and Rao-Scott adjusted chi-square tests for categorical variables. PIR: poverty-to-income ratio.

### Independent associations of vitamin B2 and vitamin E intake with cognitive function

**Figure 2:**
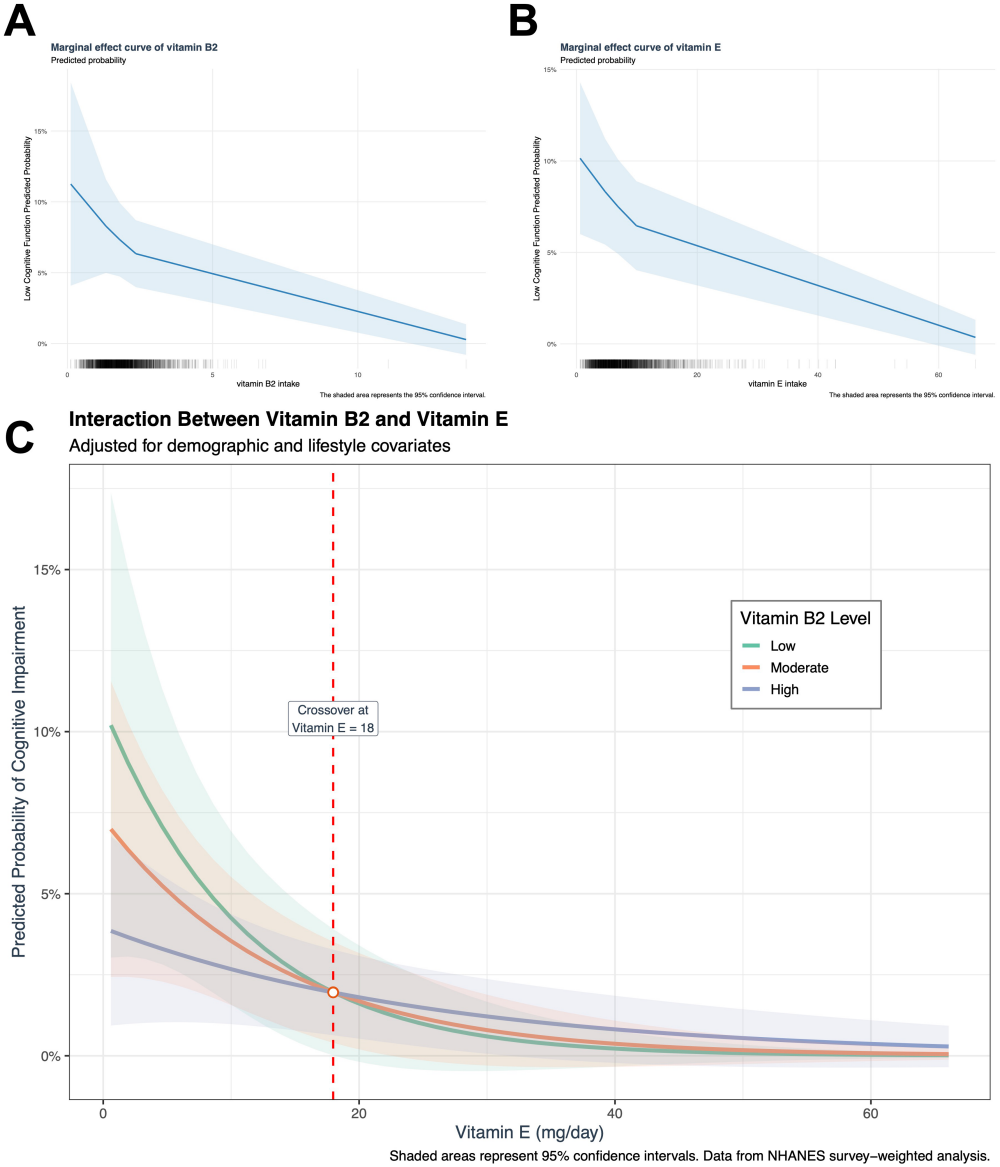
Dose-dependent interaction between dietary vitamin B2 and E intake on cognitive performance. Adjusted marginal effects derived from multivariable logistic regression models: **(A)** Association between vitamin B2 intake and probability of low cognitive function (DSST <33) in the joint effect model. **(B)** Association between vitamin E intake and predicted probability of low cognitive function in the joint effect model. **(C)** A dose-dependent interaction has been observed between vitamin E and vitamin B2. The analysis of interaction effects was conducted using pre-specified vitamin B2 intake percentiles (low: The 10th percentile was found to be 0.995 mg/day; the moderate was found to be 50th percentile, 1.799 mg/day; and the high was found to be 90th percentile, 3.029 mg/day). The presence of vertical dashed lines indicates the interaction threshold (vitamin E 18 mg/day). In the range below the established threshold (vitamin E < 18 mg/day), a synergistic protective effect was observed. This indicates that, at a constant vitamin E intake level, an increase in vitamin B2 intake resulted in a stronger cognitive function protection. This suggests that the combined antioxidant capacity of the two vitamins exceeds the sum of the individual effects of each component. Conversely, above the threshold (vitamin E ≥ 18 mg/day), an antagonistic interaction was observed, characterized by a weakening of the cognitive function protection as vitamin B2 intake increased. At equivalent levels of vitamin E intake, a lower intake of vitamin B2 was associated with higher cognitive function protection, suggesting that the antioxidant capacity was below the anticipated additive effect.

After adjusting for socio-demographic, lifestyle, and clinical confounders, high intakes of dietary vitamin B2 (OR = 0.74, 95%CI: 0.58–0.95) and vitamin E (OR = 0.73, 95%CI: 0.56–0.94) were independently associated with a lower risk of low cognitive function (DSST<33) (both *p* < 0.05) (Table 2). The two types of vitamins showed similar protective associations in the independent models, suggesting that they may affect cognitive function through different mechanisms. This is also supported by the separate marginal effect plots for vitamin B2 and vitamin E (Figures 2A,B).

**Table 2:** Independent, joint, and interactive associations of dietary vitamin B2 and E intake with low cognitive function.

Variable	Model 1 Independent Model OR 95%CI	Model 2 Joint Main Effects Model OR 95%CI	Model 3 Interaction Model OR 95%CI
Vitamin B2 (Per 1 SD)	0.74 (0.58–0.95) ^*^	0.78 (0.60–1.00) ^*^	0.77 (0.60–0.97) ^*^
Vitamin E (Per 1 SD)	0.73 (0.56–0.94) ^*^	0.76 (0.58–0.99) ^*^	0.69 (0.51–0.92) ^*^
Vitamin B2 × Vitamin E	–	–	1.15 (1.05–1.26) ^**^

Odds ratios (OR) and 95% confidence intervals (CI) from multivariable logistic regression models. All models adjusted for covariates including age, gender, education, PIR, smoking, alcohol use, physical activity, sleep duration, BMI, diabetes, hypertension, CVD, dietary fiber, and total energy intake. Interaction terms were modeled as continuous variables (per 1-SD increase). *p < 0.05; **p < 0.01; ***p < 0.001.

### The combined effect and interaction of vitamins B2 and E

In the joint model that included both vitamins, the protective associations of vitamin B2 (OR = 0.78, 95%CI: 0.60–1.00) and vitamin E (OR = 0.76, 95%CI: 0.58–0.99) were slightly weakened, but remained statistically significant. The interaction model further revealed an antagonistic effect: the B2 × E interaction term OR = 1.15 (95% CI: 1.05–1.26, *p* < 0.01), indicating that high doses of vitamin E may weaken the protective effect of vitamin B2 (Table 2).

### Stratified validation of dose-dependent interactions

Marginal effect analysis showed that when vitamin E intake was below 18 mg/day, there was a synergistic effect between vitamin B2 intake and vitamin E intake, but when vitamin E ≥ 18 mg/day, this effect reversed, and an increase in B2 intake may reduce the protective effect of vitamin E. However, from the marginal benefit graph, it can be seen that when the intake of vitamins B2 and E increases to a certain level, the protective effect of the two tends to stabilize (Figure 2C). Meanwhile, joint quartile subgroup analysis showed that compared to the double-low intake group, the high B2 + low E group (OR = 0.56, 95% CI: 0.32–0.98) and the double-high intake group.

(OR = 0.44, 95% CI: 0.27–0.73) were negatively associated with the risk of low cognitive function (Table 3), and this negative association was stronger than that of individual nutrients. There was no significant difference in the low B2 + high E group (OR = 0.69, 95% CI: 0.39–1.23) (Table 3; Supplementary Figure S1). Consistent with these findings, the “low-B2 + low-E” group (39.40%) had the highest prevalence of low cognitive function, followed by the “high-B2 + low-E” group (28.37%), the “low-B2 + high-E” group (24.22%), and the “high-B2 + high-E” group (16.22%) (Table 3; Supplementary Figure S1).

**Table 3:** Stratified associations of combined vitamin B2 and E intake quartiles with cognitive decline risk.

Intake combinations	No. of cases/sample (%)	Model 1 OR 95%CI	Model 2 OR 95%CI	Model 3 OR 95%CI
Low Vitamin B2 + Low Vitamin E	132/335 (39.40)	1.00 (Ref.)	1.00 (Ref.)	1.00 (Ref.)
Low Vitamin B2 + High Vitamin E	70/289 (24.22)	0.50 (0.28–0.87) ^*^	0.68 (0.39–1.21)	0.69 (0.39–1.23)
High Vitamin B2 + Low Vitamin E	82/289 (28.37)	0.45 (0.27–0.77) ^**^	0.57 (0.33–0.99) ^*^	0.56 (0.32–0.98) ^*^
High Vitamin B2 + High Vitamin E	257/1584 (16.22)	0.26 (0.18–0.39) ^***^	0.45 (0.27–0.75) ^**^	0.44 (0.27–0.73) ^**^
*P* for trend	-	<0.001	0.002	0.001

Vitamin intake categories (high/low) were defined by population-based quartiles. Adjusted ORs (95% CI) were calculated across three nested models: Model 1: Adjusted for age, gender, education, and PIR. Model 2: Additional adjustment for smoking, alcohol use, physical activity, sleep duration, dietary fiber, and total energy. Model 3: Fully adjusted for all covariates, including BMI, diabetes, hypertension, and CVD. Trend p-values (p for trend) were calculated using the Cochran-Armitage test. *p < 0.05; **p < 0.01; ***p < 0.001.

## Discussion

### Significance of key findings

This study provides the first evidence of a dose-dependent interaction between dietary vitamin B2 and E in modulating cognitive function among older adults. Below a threshold of 18 mg/day vitamin E intake, the two nutrients exhibit synergistic protective effects, whereas their interaction transitions to antagonism above this level. This discovery resolves inconsistencies observed in prior trials, where isolated high-dose vitamin E supplementation failed to reduce dementia risk (27) and B-vitamin regimens showed minimal cognitive benefits—discrepancies potentially attributable to unaccounted nutrient interactions. Our findings challenge the conventional single-nutrient paradigm and emphasize the necessity of evaluating combinatorial nutrient exposures in aging populations.

### Potential mechanisms underlying the interaction

The transition from synergy to antagonism likely reflects dynamic redox equilibrium modulated by vitamin E levels. At intakes below 18 mg/day, *α*-tocopherol’s lipid peroxidation inhibition cooperates with vitamin B2-mediated FAD-dependent glutathione recycling to amplify antioxidant defenses (28–30). However, therapies interfering with FAD synthesis, such as tocopherol-derived PMCol, may disrupt this synergy (31). High-dose α-tocopherol supplementation metabolizes to tocopherol-derived compounds like PMCol, which competitively inhibits riboflavin kinase—the enzyme converting dietary vitamin B2 to FAD. This interference appears to be dose-dependent and primarily relevant to supplemental intake (>18 mg/day), where PMCol accumulation exceeds physiological levels. In contrast, dietary vitamin E from whole foods (e.g., nuts, seeds) rarely achieves such concentrations, minimizing PMCol formation and preserving FAD synthesis. Beyond the threshold, three interrelated mechanisms may drive antagonism: gut microbiota-mediated suppression of B2-to-FAD conversion (8, 32), vitamin E-induced oxidative DNA damage counteracting B2’s genomic stabilization (33, 34), and pro-oxidant effects of excess *α*-tocopherol accelerating B2 catabolism (35–37). While these pathways align with the interconnected roles of B2 in redox cofactor supply and E in lipid antioxidant activity, direct molecular validation remains essential.

### Clinical and public health implications

Clinically, our stratified analyses revealed robust cognitive protection in both high-B2 + low-E (OR = 0.56) and dual-high intake groups (OR = 0.44). However, marginal effects identified 18 mg/day as a critical threshold: above this level, reduced B2 intake unexpectedly enhanced protection. This paradox challenges universal supplementation paradigms, advocating instead for precision nutrient ratios tailored to baseline intake. For older adults with vitamin E exceeding 18 mg/day (common among supplement users), moderating B2 intake may optimize neuroprotection—a hypothesis requiring verification through targeted trials. Current guidelines should integrate such thresholds to refine dietary counseling, prioritizing B2-E balance over isolated high-dose regimens.

### Limitations and future directions

Several limitations temper interpretation. First, the cross-sectional design precludes inferences about temporality or causality. Reverse causation remains a significant concern, as cognitive decline may itself alter dietary habits (e.g., reduced intake of nutrient-dense foods), potentially biasing observed associations. Although we adjusted for sociodemographic and health confounders, longitudinal studies are essential to clarify directionality. Second, although interviewers were rigorously trained in the standardized protocol, potential minor heterogeneity in interviewer probing techniques could introduce measurement error in dietary recall data. Third, while NHANES dietary data quantifies total vitamin E without distinguishing isoforms, our mechanistic interpretation specifically concerns *α*-tocopherol—the primary form in supplements and the isoform metabolized to PMCol. As *γ*-tocopherol constitutes >70% of dietary vitamin E in nuts/seeds yet exhibits weaker riboflavin kinase inhibition, the observed antagonism likely reflects high-dose α-tocopherol exposure rather than total vitamin E intake. This distinction reinforces that our findings are most applicable to supplemental α-tocopherol contexts. Furthermore, the potential impact of dietary supplements should have been considered, yet this was precluded by the unrecorded data concerning vitamin E supplements. Future studies should employ longitudinal designs to establish temporal relationships and incorporate biomarkers (plasma FAD, urinary γ-CEHC) to clarify bioactive nutrient fractions. Randomized trials comparing combinatorial regimens (e.g., high-B2 + low-E vs. balanced ratios) are urgently needed to translate findings into clinical practice.

## Conclusion

In conclusion, our study identifies a dose-dependent threshold governing dietary vitamin B2-E interactions in cognitive protection. Below 18 mg/day vitamin E intake, synergy predominates, whereas antagonism emerges beyond this level—a critical nuance absent in current guidelines. Personalized strategies optimizing B2-E ratios may outperform isolated high-dose supplementation, particularly in older adults with preexisting elevated vitamin E intake. While mechanistically plausible, causal validation through biomarker-integrated longitudinal studies and trials remains imperative. Future work must also explore gut microbiota’s role in modulating these interactions and assess whether threshold-aware dietary strategies delay dementia onset, advancing precision nutrition in cognitive aging.

## Data Availability

The datasets presented in this study can be found in online repositories. The names of the repository/repositories and accession number(s) can be found at: https://wwwn.cdc.gov/nchs/nhanes/default.aspx.
