# Supplementary figures and images for: Dose-dependent interaction of dietary vitamin B2 and E in relation to cognitive performance: a cross-sectional study of older adults
Source: Front Nutr. 2025 Sep 10;12:1597724. doi: 10.3389/fnut.2025.1597724 (PMC12457415; doi:10.3389/fnut.2025.1597724)

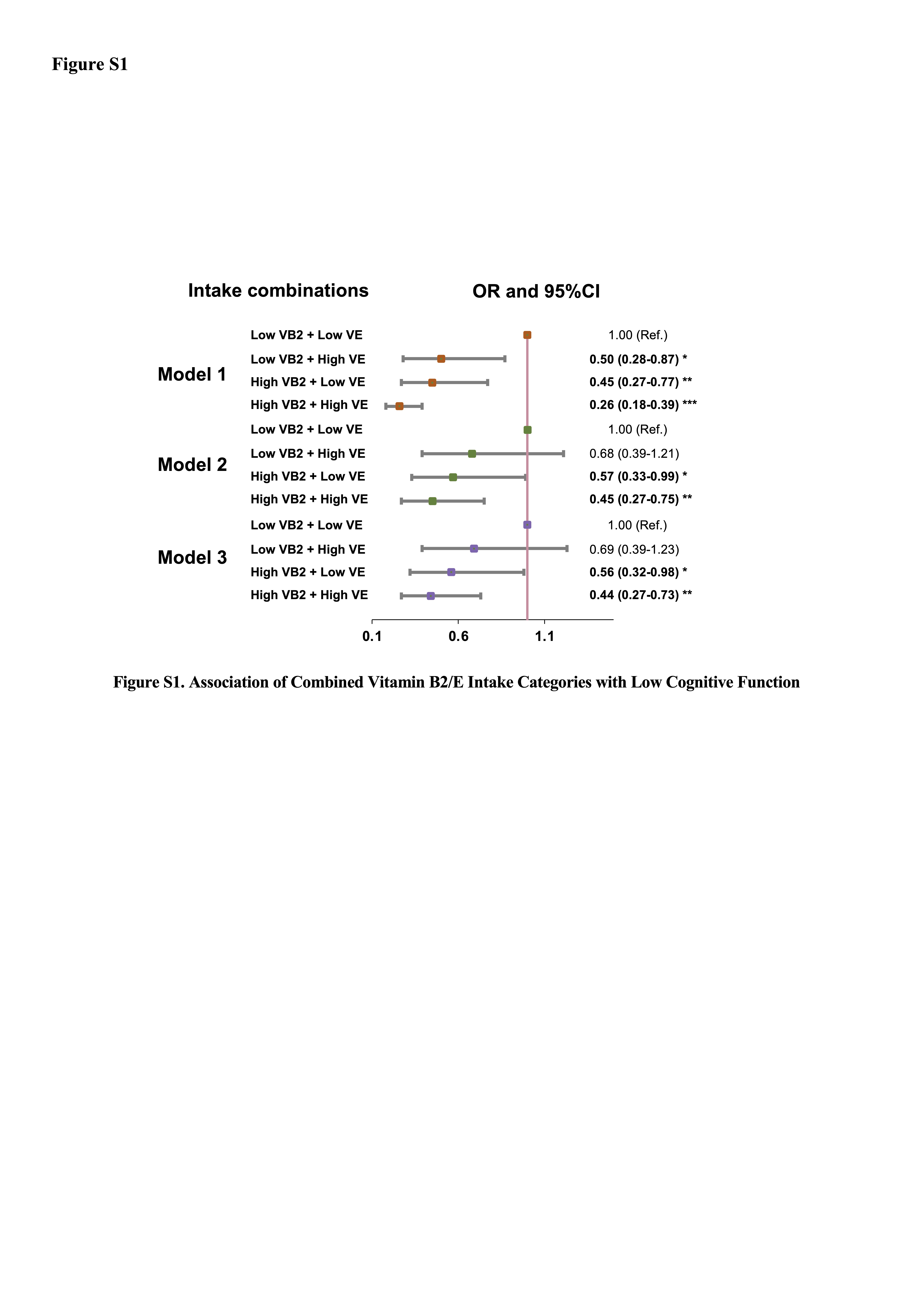

Supplement: Supplementary file 1 [file Image_1.jpg]
